# Gold Nanoparticles Enhance EGFR Inhibition and Irradiation Effects in Head and Neck Squamous Carcinoma Cells

**DOI:** 10.1155/2020/1281645

**Published:** 2020-11-07

**Authors:** Masahiko Kashin, Yasumasa Kakei, Shun Teraoka, Takumi Hasegawa, Akinobu Yamaguchi, Takao Fukuoka, Ryohei Sasaki, Masaya Akashi

**Affiliations:** ^1^Department of Oral and Maxillofacial Surgery, Kobe University Graduate School of Medicine, Kobe 650-0017, Japan; ^2^Laboratiory of Advanced Science and Technology for Industry, University of Hyogo, Kamigori 678-1205, Japan; ^3^Graduate School of Engineering, Kyoto University, Kyoto 615-8540, Japan; ^4^Division of Radiation Oncology, Kobe University Graduate School of Medicine, Kobe 650-0017, Japan

## Abstract

Cetuximab, an epidermal growth factor receptor inhibitor (EI), is currently the only targeted molecular therapy used in combination with radiotherapy for head and neck squamous cell carcinoma (HNSCC). Gold nanoparticles (AuNPs) are expected to enhance radiotherapy effects in cancers. To investigate whether AuNPs combined with AG1478, an EI, enhanced irradiation effects on HNSCC cells, we first examined AG1478 adsorption on AuNP surfaces, using surface-enhanced Raman scattering, which indicated an adsorption equilibrium of AG1478 to AuNPs. We then used transmission electron microscopy to find internalization rates of AuNP alone and AuNP+AG1478; we found that intracellular uptake of AuNP alone and AuNP+AG1478 did not significantly differ. We compared cell numbers, proliferation, apoptosis, and migration between control cells and those treated with or without 60 nm AuNP (1.0 nM), AG1478 (0.5 *μ*M), and irradiation (4 Gy). We found that AuNP+AG1478 inhibited proliferation more than AG1478 alone; the combination of irradiation+AuNP+AG1478 significantly reduced total cell numbers compared with the combination of irradiation+AuNP; AuNP+AG1478 increased apoptotic reaction to irradiation; the combinations of AuNP+AG1478 and irradiation+AuNP induced more apoptosis than AG1478+irradiation. Whereas AuNP+AG1478 enhanced cytotoxicity in human HNSCC cells by inhibiting proliferation, irradiation+AuNP enhanced cytotoxicity by inducing apoptosis.

## 1. Introduction

Over 90% of all head and neck cancers are squamous cell carcinomas, and 90%–100% of head and neck squamous cell carcinomas (HNSCCs) overexpress epidermal growth factor receptor (EGFR) [1]. Overexpression of EGFR is correlated with decreased overall and disease-free survival and increased resistance to radiation therapy (RT) and locoregional relapse [2–4].

EGFR inhibitors (EIs) have been tested against HNSCC in several randomized clinical trials. In 2006, cetuximab, a chimeric antibody (Ab) that targeted EGFR, was included in HNSCC regimens after a phase III trial and showed a survival benefit for cetuximab+RT compared with RT alone [5]. Since 2008, the regimen of cetuximab+platinum +5-fluorouracil has been the first-line standard of care for HNSCC, based on a randomized trial [6]. Although cetuximab enhanced the effects of RT and chemotherapy in these studies, results were disappointing, as only 10%–30% of patients responded to monotherapy. This implies some intrinsic resistance [7]. A novel strategy to improve response by EGFR^+^ HNSCC is therefore needed.

Recently, nanoparticle- (NP-) based approaches have been investigated for improving cancer response to both RT and chemotherapy [8]. Noble metal NPs have been introduced as novel drug delivery systems for anticancer therapies [9–12]. Gold nanoparticles (AuNPs) have attracted particular interest as they are easy to synthesize in various sizes and shapes and to functionalize with biomolecules that, together with their particular optical properties, may have both therapeutic and diagnostic applications in oncology [13]. Additionally, because of gold's high atomic number and well-established biosafety, AuNPs may be suitable as radiosensitizing agents [14–17].

AuNPs easily combine with many biological ligands, such as single antibodies or peptides, which can be used as probes to selectively recognize cancer [18, 19]. Thus, AuNPs could potentially be functionalized with anti-EGFRAb to treat EGFR-overexpressing tumors such as HNSCCs. Anti-EGFR monoclonal Ab (mAb; EGFRmAb) conjugated to AuNPs (EGFRmAb–AuNP) can reportedly induce apoptosis in squamous laryngeal and breast cancer cell lines [20, 21]. However, as the effects of radiation+EGFRmAb–AuNP in HNSCC cells are unclear, we used cell counting, proliferation, and wound healing assays and apoptosis counts to evaluate this combination on HNSCC cells; surface-enhanced Raman scattering (SERS) to study the adsorption of EI+AuNP; and transmission electron microscope (TEM) analysis to assess the uptake ratio of EI+AuNP.

## 2. Materials and Methods

### 2.1. Cell Culture

We used the human HNSCC cell line HSC-3 (tongue carcinoma; Japanese Collection of Research Bioresources Cell Bank, Tokyo, Japan). The cells were cultured in Dulbecco's modified Eagle's medium (DMEM; Wako Pure Chemical Industries, Ltd., Osaka, Japan) supplemented with 10% (v/v) fetal bovine serum (Biowest, Nuaillé, France) and 1% penicillin/streptomycin (Sigma-Aldrich; Merck KGaA, Darmstadt, Germany) at 37°C in a humidified atmosphere of 5% (v/v) CO_2_. Cells were incubated in DMEM for 48 h prior to treatment. We used cells that were 2–10 passages from their primary culture.

### 2.2. Reagents and Antibodies

Citrate-stabilized AuNPs were purchased from Cytodiagnostics, Inc. (Burlington, ON, Canada). Standard AuNPs (60 nm) were used (lot number: 2458052_60). Rat anti-E-cadherin mAb (ECCD2) was purchased from Takara Bio, Inc. (Otsu, Japan), and the tyrosine kinase inhibitor, AG1478, was obtained from Merck KGaA (Darmstadt, Germany). Secondary antibodies conjugated with Cy3 donkey anti-mouse IgG (H+L; AP192C) were purchased from Invitrogen (Thermo Fisher Scientific, Inc., Waltham, MA, USA). Cleaved caspase-3 Ab was purchased from Cell Signaling Technology (Danvers, USA).

### 2.3. Detection of Adsorption of AG1478 on AuNPs

We used SERS on the tyrosine kinase inhibitor AG1478 in solution with AuNP colloids to determine whether AG1478 is adsorbed on the surfaces of AuNPs. In SERS, the intensity of Raman scattering is greatly amplified when a molecule is adsorbed on a rough surface, such as a precious metal, compared with bulk molecules, by a factor of 10^4^–10^6^ [22, 23]. In 1997, Nie et al. made it possible to use SERS spectroscopy to measure a single protein molecule with AuNPs, which was a dramatic step forward in this technique [24]. SERS techniques require a noble metal nanostructure. Gold or silver nanoparticles are usually used because they are capable of excitation of the essentially strong localized surface plasmon resonance; AuNPs, in particular, are also chemically stable, generally inert, and are easy to use [25].

For the AuNPs, we used a 60 nm gold colloid, reduced with citric acid. AG1478 (5 mg) was dissolved in 1 mL ethanol for a solution of ~15.8 mM and diluted sequentially with ethanol to make AG1478 solutions of 1 mM, 100, 10, 3, 2, 1 *μ*M, and 500 nM concentrations in 1 mL Eppendorf tubes. In an approximately 100 *μ*L capacity aluminum pan, we mixed AG1478 ethanol solution (3 *μ*L), 1 M NaCl (3 *μ*L), and AuNP (54 *μ*L) by pipetting [26, 27] and coaggregated the mixture to generate self-assembled anisotropic structures with SERS activity. Coaggregation allows the absorption spectra of the liquid to converge to the same shape, so that the electric field enhancement by localized plasmon resonance will be almost the same and quantitative measurement of intensity of Raman scattering can be expected [28, 29]. Additionally, the measurement can be stable for about 10 min until the agglomeration settles down. Concentration of this solution becomes 5%. It was examined with a simple Raman spectroscope, RAM100S (Lambda Vision), equipped with a laser of 785 nm (100 mW). The solution was placed in an aluminum pan and exposed, 10 times, to radiation for 0.1 s or 1 s. All experiments were performed at room temperature.

### 2.4. TEM Images of Cellular AuNP Uptake

To determine whether binding AuNPs to AG1478 affected AuNP uptake, lysosomal uptake of AuNP was measured by TEM. HSC-3 cells were cultured with 60 nm AuNPs (50 nM), both alone and as a mixture of 60 nm AuNP (50 nM) and AG1478 (0.5 *μ*M). The samples were fixed with 2% paraformaldehyde and 2% glutaral aldehyde (GA) in 0.1 M phosphate buffer (PB) pH 7.4 at incubation temperature and put into a refrigerator for 30 min to lower the temperature to 4°C. Thereafter, they were fixed with 2% GA in 0.1 M PB at 4°C overnight. After fixation, the samples were washed three times with 0.1 M PB for 30 min each and postfixed with 2% osmium tetroxide (OsO_4_) in 0.1 M PB at 4°C for 1 h. The samples were dehydrated in an alcohol gradient, then transferred to a resin (Quetol-812; Nisshin EM Co., Tokyo, Japan), and polymerized at 60°C for 48 h. The polymerized resins were sectioned at 80 nm with a diamond knife, using an ultramicrotome (Ultracut UCT; Leica, Vienna, Austria), mounted on copper grids, stained with 2% uranyl acetate for 15 min, washed with distilled water, and secondary stained with lead stain solution (Sigma-Aldrich Co., Tokyo, Japan) for 3 min. The grids were visualized by a TEM (JEM-1400Plus; JEOL Ltd., Tokyo, Japan) at an acceleration voltage of 100 kV. Digital images (3296 × 2472 pixels) were taken with a CCD camera (EM-14830RUBY2; JEOL Ltd.).

### 2.5. Gold Nanoparticles, AG1478, and Irradiation

Cells were grown in 2 mL DMEM on glass slips in 35 × 10 mm polystyrene tissue culture dishes at a density of 1 × 10^5^ cells/mL. Cells were incubated with AuNPs, diluted with 1.0 nM phosphate-buffered saline (PBS) and AG1478 at 0 or 0.5 *μ*M. After 24 h at 37°C, samples were irradiated by a MBR-1505R2 X-ray generator (Hitachi, Tokyo, Japan) at 150 kV, 4 mA, using a 1 mm aluminum filter. Cells were exposed to a fixed dose of 4 Gy, as reported previously [30]. Cells were incubated at 37°C for 48 h, after which experiments were conducted.

### 2.6. Cell Counting by Immunofluorescence Staining and Microscopy

Cells were grown with DMEM on glass slips in 35 × 10 mm polystyrene tissue culture dishes at a density of 1 × 10^5^ cells/mL for 24 h. We added AG1478 (0 [control] or 0.5 *μ*M) and AuNPs (0 [control] or 1 nM) to each well. After 24 h incubation, cells were irradiated. After 48 h irradiation, we fixed with 1% formaldehyde in PBS for 10 min, treated with 0.2% Triton X-100 in PBS for 5 min and washed with PBS, then blocked with 1% bovine serum albumin (BSA; Sigma-Aldrich; Merck KGaA) in PBS for 30 min and incubated with primary antibodies (rat anti-E-cadherin mAb) for 24 h at 4°C. All antibodies were diluted with 1% BSA in PBS, then rinsed three times with PBS and stained with 4′,6-diamidino-2-phenylindole (DAPI; Thermo Fisher Scientific, Inc., Waltham, MA, USA) and incubated with corresponding secondary antibodies (1 : 500; Cy3®) for 30 min. After rinsing with PBS, specimens were embedded in FluorSave™ (Merck KGaA). They were then viewed with a BZ-X 700 fluorescence microscope (Keyence Corporation, Osaka, Japan). Cell numbers were calculated by counting DAPI^+^ nuclei; to count cell junctions, numbers of cell–cell borders in which E-cadherin was expressed were counted in 10 randomly selected microscopic images (magnification ×20). Results were expressed as averaged data of three independent experiments.

### 2.7. Apoptosis

To detect apoptotic cells, cells were grown with DMEM on glass slips in 35 × 10 mm polystyrene tissue culture dishes at a density of 1 × 10^5^ cells/mL for 24 h. We added AG1478 (0 [control] or 0.5 *μ*M) and AuNPs (0 [control] or 1 nM) to each well. After a 24-hour incubation, cells were irradiated. Forty-eight hours after the irradiation, we fixed with 1% formaldehyde in PBS for 10 min, treated with 0.2% Triton X-100 in PBS for 5 min and washed with PBS, then blocked with 1% bovine serum albumin (BSA; Sigma-Aldrich; Merck KGaA) in PBS for 30 min and incubated with primary antibodies (rat anti-E-cadherin mAb) for 24 h at 4°C. All antibodies were diluted with 1% BSA in PBS. The cells were then rinsed three times with PBS and stained with caspase-3 (Cell Signaling Technology, Danvers, USA) and 4′,6-diamidino-2-phenylindole (DAPI; Thermo Fisher Scientific, Inc., Waltham, MA, USA) and incubated with corresponding secondary antibodies (1 : 500; Cy3®) for 30 min. After rinsing with PBS, specimens were embedded in FluorSave™ (Merck KGaA) and observed under a fluorescence microscope. Apoptotic cells were calculated as a percentage of total cells.

### 2.8. Cell Survival

Growth inhibitory effects of AuNP, AG1478, and irradiation were evaluated. Numbers of viable cells were determined by using a Cell Counting Kit-8 (CCK-8; Dojindo Molecular Technologies, Inc., Kumamoto, Japan) according to the manufacturer's instructions. We suspended 5 × 10^3^ cells in complete medium, in a 96-well plate (100 *μ*L/well) at 37°C. After 24 h, we added AG1478 and/or AuNPs at the above-described concentrations to each well. After a 24-hour incubation, cells were irradiated. We added CCK-8 solution (10 *μ*L) to cells 48 h after irradiation. Plates were then incubated for 1–4 h, after which absorbance was measured at 450 nm using a microplate reader.

### 2.9. Wound Healing Assay

Cells (3 × 10^5^ cells/mL) were plated in duplicate in 6-well plates, grown to 70%–80% confluence, and incubated with high-glucose DMEM that contained AG1478 (0 [control] or 0.5 *μ*M) and AuNPs (0 [control] or 1 nM). After 24 h, cells were exposed to irradiation (0 [control] or 4 Gy). After 48 h, cell monolayers were scraped with sterile 200 *μ*L disposable plastic pipette tips and washed with PBS. Wound healing was checked with microscopy at 0, 4, 8, 12, 16, 20, and 24 h at 37°C after wounding. Images were taken with a BZ-X700 fluorescence microscope (Keyence Corporation; 4x magnification), and area was calculated by an analysis software BZ-H4M (Keyence).

### 2.10. Statistical Analysis

Each of our experiments was performed in triplicate with results expressed as mean ± standard deviation. Statistical analyses were performed using the R software (R Development Core Team, 2011). One-way analysis of variance and Tukey–Kramer multiple comparison tests were used on cell counting, cell survival, and apoptosis data. *P* < 0.05 was considered significant.

## 3. Results

### 3.1. Comparison of Solid Raman Spectra

**Figure 1:**
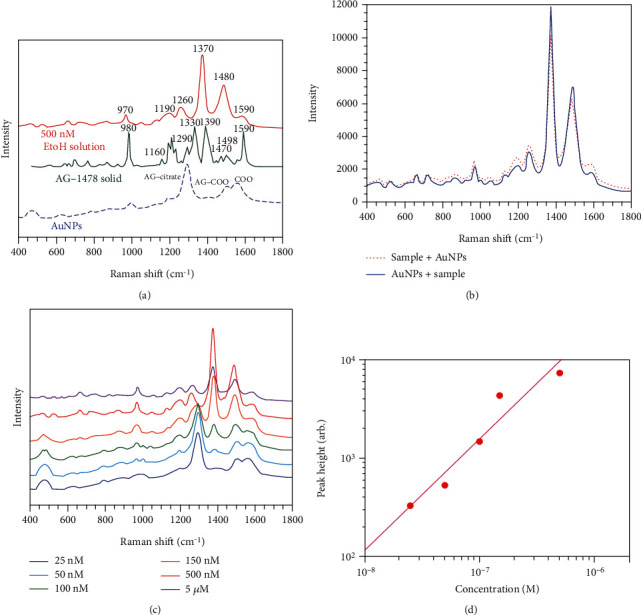
(a) Enhanced Raman spectrum of AG-1478 500 nM EtOH/solution, normal Raman spectrum of solid, and citrate-capped AuNPs. (b) Enhanced Raman spectrum when the order of mixing AG-1478 500 nM EtOH/solution and AuNPs was changed from front to back. (c) Enhanced Raman spectra of AG1478 concentrations at 5 *μ*M, 500, 150, 100, 50, and 25 nM in the measured solution. (d) [Log]–[log] plot of the SERS peak height at 1370 cm^−1^ by concentration yielded nearly sigmoid-like calibrations in the range of 500, 150, 100, and 50 nM AG1478.

We initially used SERS measurements to see whether AG1478 is adsorbed on the surfaces of AuNPs. First, we checked the Raman spectrum of AG1478 in its solid state and in its liquid phase with citric acid-reduced AuNPs.  Figure 1(a) shows the comparison of Raman spectra of citrate-capped AuNPs without AG1478 (blue dashed line), 500 nM AG1478 in ethanol solution with AuNPs (red solid line), and spontaneous Raman of AG1478 solid (green solid line). As shown in Figure 1(a), when the citrate-capped AuNPs alone were aggregated, peaks of citric acid adsorbed on the surface appeared at 1580 cm^−1^ (unadsorbed citrate ion C=O stretching asymmetrically in COO^−^), 1535 cm^−1^ (adsorbed citrate ion Au-COO^−^ stretching), and 1295 cm^−1^ (adsorbed citrate ion [blue dashed line]), whereas a clearly different spectrum (red line) was observed in the 500 nM AG1478 solution, in which the main peaks appeared at 1590, 1480, 1370, 1260, 1190, and 970 cm^−1^. However, in measuring the Raman spectra of AG1478 solids with another Raman microspectrometer (green line), the main peaks appeared at 1590, 1498, and 1470; 1390, 1330, and 1290; 1160; and 980 cm^−1^. By comparing with these spectra, the main peak positions of AG1478 in ethanol (red solid line) are expressed near those of solids and are different from the Raman spectrum of the citrate-treated AuNPs. As the spectrum shape is slightly changed from that of the solid because it is dissolved in ethanol, the spectrum observed for the 500 nM solution (red solid line) in the presence of AuNPs was considered to be the SERS spectrum derived from AG1478.

### 3.2. Sample Mixing Order

AG1478 is a water-insoluble molecule that may adsorb to the aluminum of its container. Therefore, we tried adding AuNPs to an aluminum pan first, followed by the AG1478 ethanol solution, and 1 M NaCl in that order. At 500 nM, the spectra were almost the same whether AuNPs were added first or later (Figure 1(b)). However, adding AG1478 before adding NaCl caused aggregation, which allowed for the formation of self-assemblies with the same localized plasmon resonance. This aggregation induced by AG1478 can affect the formation of highly sensitive SERS-active self-assemblies induced by the coaggregation method. Therefore, we continued to add AuNPs later in the following experiments.

### 3.3. SERS and Calibration Curves for Ethanol Solution

Spectra of AG1478 concentrations at 5 *μ*M, 500, 150, 100, 50, and 25 nM in the measured solution are shown in Figure 1(c). From 5 *μ*M to 50 nM, characteristic peaks appeared at 1480, 1370, 1260, and 970 cm^−1^, depending on the concentration. At concentrations below 500 nM, the peak at 1295 cm^−1^ from the citric acid adsorbed on the AuNPs was stronger. At 25 nM, almost no peak from AG1478 could be observed. A [log]–[log] plot of the SERS peak height at 1370 cm^−1^ by concentration yielded nearly sigmoid-like calibrations in the range of 500, 150, 100, and 50 nM (Figure 1(d)). The appearance of enhanced citric acid peaks at low concentrations, and this sigmoidal behavior suggests an adsorption equilibrium between AG1478 and AuNPs.

### 3.4. Intracellular Uptake of AuNPs by Adding AG1478

**Figure 2:**
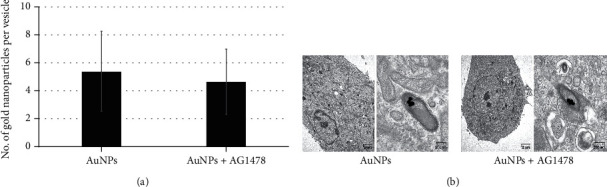
(a) Adding AG1478 did not increase numbers of AuNPs in cluster-forming vesicles. The actual amount of AuNP uptake did not significantly differ between the AuNP-alone and AG1478+AuNP groups. (b) Representative TEM images. AuNP clusters consisted of several AuNPs.

TEM showed dispersed gold particles in endocytic vesicles, but none were detected in the nucleus. Numbers of AuNPs in vesicles that formed clusters did not significantly differ between the AuNP-alone and AG1478+AuNP groups (Figure 2(a)).  Figure 2(b) shows representative TEM images. AuNP clusters consisted of several AuNPs.

### 3.5. Cell Counting Assay

**Figure 3:**
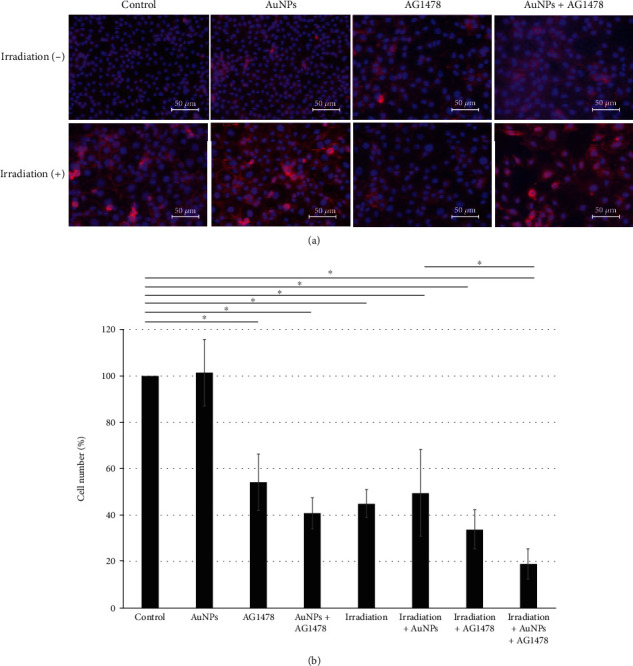
(a) Effects of the combination of 1 nM AuNP+0.5 *μ*M AG1478 on HNSCC cell proliferation, with or without 4 Gy irradiation. For counting, cells and their nuclei were visualized using DAPI and E-cadherin. (b) Compared with the control group and the AuNP-alone group, the other six groups all showed significantly decreased cell numbers. In addition, the 0.5 *μ*M AG1478+1 nM AuNP+4 Gy irradiation group had significantly lower cell counts than the 1 nM AuNP+4 Gy irradiation group.

We assessed the effects of three concentrations of 60 nm AuNPs without X-rays in HSC-3 cells. Cell numbers did not significantly differ between the control cells and those treated with 1.0, 10, or 50 nM AuNPs (data not shown). Representative immunohistochemistries of eight groups, with or without 1 nM AuNP, 0.5 *μ*M AG1478, and 4 Gy irradiation, are shown in Figure 3(a).

Compared with the control group and the AuNP-alone group, the other six groups all showed significant decreases in cell numbers (Figure 3(b)). Moreover, the 0.5 *μ*M AG1478+1 nM AuNP+4 Gy irradiation group had significantly lower cell counts than the 1 nM AuNP+4 Gy irradiation group (*P* < 0.001; Figure 3(b)).

### 3.6. Proliferation Assay

**Figure 4:**
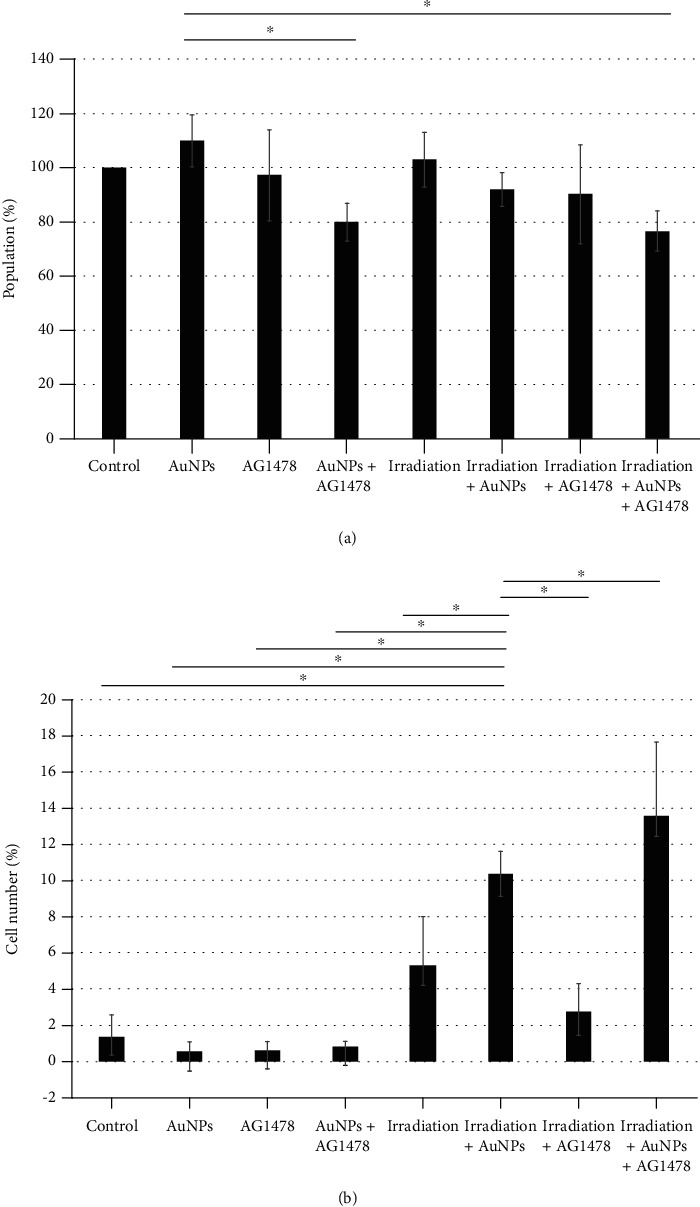
(a) Adding AuNPs and AG1478 and/or irradiation significantly inhibited cell proliferation. Cell Counting Kit-8 assay shows the combination of 1 nM AuNP+0.5 *μ*M AG1478 inhibited HNSCC cell proliferation, both with and without 4 Gy irradiation. *Y*-axis values indicate percentages, with the control as 100%. Data represent three independent experiments and are presented as means ± standard deviation. AuNPs: gold nanoparticles. (b) Adding AuNPs and/or AG1478 significantly enhanced the apoptotic effect of irradiation. The effects of combining 1 nM AuNPs+0.5 *μ*M AG1478 on apoptosis in HNSCC cells, with or without 4 Gy irradiation, significantly increased the percentage of capase-3^+^ (apoptotic) cells compared with 4 Gy irradiation alone. *Y*-axis values are expressed as percentages, with the control as 100%. The data represent three independent experiments and are presented as means ± standard deviation.

We used proliferation and apoptosis assays to establish the underlying causes of the decreased cell numbers in the irradiation+AuNP+AG1478 group. Compared with AuNP alone, the AuNP+AG1478 groups, both with irradiation (*P* = 0.02) and without irradiation (*P* = 0.03), were significantly reduced on cell growth (Figure 4(a)).

### 3.7. Measurement of Apoptosis

The combination of AuNP+irradiation induced apoptosis, as shown by the significantly greater percentage of caspase-3^+^ cells, compared with the AuNP-alone, AG1478-alone, irradiation-alone, and irradiation+AG1478 groups (*P* < 0.001; Figure 4(b)). Moreover, the AuNP+AG1478+radiation had a significantly greater apoptotic percentage compared with AuNP+irradiation (*P* < 0.001; Figure 4(b)).

### 3.8. In Vitro Wound Healing Assay

**Figure 5:**
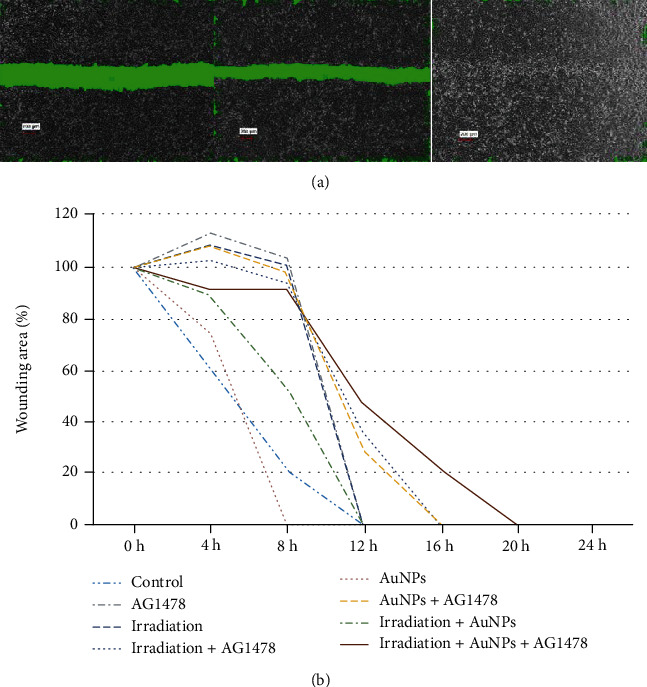
(a) Representative migration images. The scratched area was calculated from the area enclosed in green, which was automatically analyzed by Keyence's software BZ-H4M, according to the area of the central part of the scratch, excluding the missing part of the cells. (b) Adding AuNPs and/or irradiation to AG1478 reduced cell motility compared with AG1478 only. *Y*-axis values are expressed as percentages, with the control as 100%. The data represent three independent experiments and are presented as means ± standard deviation.

In Figure 5(a), the area was calculated from the area enclosed in green, which was automatically analyzed by Keyence's software BZ-H4M, according to the area of the central part of the scratch, excluding the missing part of the cell.

Biological responses to EGFR signaling are pleiotropic and can result in tumor-promoting processes, including enhanced cell motility [31]. Adding AuNP and/or irradiation to AG1478 reduced cell motility compared with AG1478 only (Figure 5(b)).

## 4. Discussion

This study revealed that AuNP+AG1478+irradiation led to the greatest reduction of HSC-3 cell numbers among the eight possible combinations of these treatments. Whereas reduction of cell numbers by AuNPs adsorbed on AG1478 was caused by suppression of cell proliferation, reduction of cell numbers by radiation+AuNP was caused by apoptosis.

To our knowledge, this is the first study of radiosensitization by AuNP adsorption on an EI against human HNSCC cells. However, Dahai et al. showed radiosensitization using silver nanoparticles combined with EI in nasopharyngeal carcinoma [32].

AuNPs show promise for clinical applications and may potentially be used for RT and photodynamic therapy because of their biostability, ease of surface modification, and radiation sensitization. In the present study, we confirmed the adsorption of AG1478 to the surface of AuNPs by Raman spectroscopy, but similar results were reported in previous reports with other EIs such as gefitinib and erlotinib [33, 34]. The chemical formula of AG1478 is similar to those of gefitinib and erlotinib, which suggests that the quinazoline ring portion is bound to the gold surface [33, 34].

The combination of AG1478+AuNP reduced cell proliferation compared with AG1478 only (Figure 4(a)); and AuNP+AG1478 reduced cell migratory activity compared with AG1478 only (Figure 5). As described in previous reports, the decreased migratory activity was probably caused by adhesion of drugs such as EGFR or HER2 inhibitors onto the surfaces of the AuNPs, which enhanced their anticancer effect compared with EGFR or HER2 inhibitor alone [35–37].

Uptake of AuNPs into HSC-3 cells by AG1478 addition was analyzed by TEM, but the AuNPs with and without AG1478 did not significantly differ (Figure 2(b)). However, Kubota et al. reported that HER2 inhibitor conjugated to AuNPs was efficiently internalized into cells, and this HER2-dependent internalization may affect the cytotoxic mechanisms of trastuzumab+AuNP [37]. As measuring gold particles smaller than 60 nm is difficult by the coaggregation method, AuNPs of 60 nm were used in this study. However, AuNPs of 40–50 nm are reportedly the most effective, and Kubota et al. [37] used 50 nm AuNPs. A previous study by Jiang et al. [38] reported that, although the AuNPs were found inside the cytoplasm in the trastuzumab—40 nm AuNP-treated cells, the AuNPs were found only on the cell surface in the trastuzumab—70 nm AuNP-treated cells. This result indicates that in cells treated with antibody+larger AuNPs (i.e., larger than 60 nm), little or no AuNPs are present in the cytoplasm. In our study, AuNP internalization into cells was confirmed with TEM, but why adding AG1478 had no effect on AuNP internalization should be addressed in the future research.

Although adding AG1478 to AuNPs did not increase apoptosis, the rate of apoptosis was increased by irradiation (Figure 4(b)). Binding AG1478 to AuNP surfaces did not apparently inhibit the radiosensitizing effect of AuNPs on apoptosis. Although increased apoptosis after radiation and AuNPs has been widely reported, including our own report [30], we found no other reports of irradiation and AuNPs bound to EIs in HNSCC. Melancon et al. reported that near-infrared laser irradiation of gold nanoshells conjugated with EIs reduced the number of A431 cells compared with irradiation of gold nanoshells alone [39]. Dahai et al. reported that combining irradiation with either AgNP or Ag/C225 reduced expression of DNA repair proteins [32]. These reports imply that EIs bound to AuNPs can impart high energy to DNA damage, which would be expected to increase cell damage and apoptosis. Another possible mechanism is radiation-induced reactive oxygen species (ROS) in the vicinity of the inhibitor-bound tumor cells.

A limitation of this study is that no factors other than cell viability and apoptosis (e.g., ROS production, oxidative stress, DNA damage, cell-cycle effects, and potential bystander effects) were evaluated. Further investigations into the effects of AuNPs conjugated to AG1478 on DNA repair, cell-cycle arrest, and autophagy in irradiation are needed. The current study showed that adding AuNPs enhanced the cytotoxicity of AG1478 and irradiation in vitro, but in vivo studies are required. AuNPs may remain in the body semipermanently, as no method for their metabolism and excretion has been established [40–42]; however, irradiation with AuNPs and EI may be a treatment option for HNSCC, especially oral cancers, as spatially focused treatment options, such as direct tumor injection and super selective arterial infusion therapy, are available.

## 5. Conclusions

AG1478 appeared to adsorb the surface of AuNPs by Raman spectroscopy. TEM images indicated the uptake of AuNPs into HSC-3 cells by AG1478 addition, but the AuNPs with and without AG1478 did not differ significantly. AuNPs can enhance the cytotoxic effects of irradiation and EGFR inhibitors against human HNSCC cells *in vitro*. This enhanced cytotoxicity was found to be caused by apoptosis with the AuNPs+irradiation combination and by inhibition of cell proliferation with the AuNPs+AG1478 combination.

## Data Availability

The data used to support the findings of this study are available from the corresponding author upon request.
